# Halo Nevus as a Self-Limited Model of Melanocyte Autoimmunity: Bridging Vitiligo, Immune Resolution, and Tumor Immunology—A Narrative Review

**DOI:** 10.3390/dermatopathology13030033

**Published:** 2026-07-16

**Authors:** Giulio Tosti

**Affiliations:** Dermato-Oncology Unit, Istituto di Ricovero e Cura a Carattere Scientifico (IRCCS), Istituto Europeo di Oncologia, 20141 Milan, Italy; giulio.tosti@ieo.it; Tel.: +39-0257489607

**Keywords:** halo nevus, Sutton nevus, vitiligo, melanocyte autoimmunity, CD8+ T cells, interferon-γ, granulysin, PD-L1, regulatory T cells, melanoma regression, immune checkpoints

## Abstract

Halo nevus (HN), also known as Sutton nevus or leukoderma acquisitum centrifugum, is a melanocytic lesion characterized by a depigmented halo surrounding a central nevus. Although HN is a benign dermatological condition, increasing evidence indicates that HN represents a dynamic in vivo model of melanocyte-directed immune response with relevant implications for autoimmunity, pigmentary disorders, melanoma regression, and tumor immunosurveillance. The pathogenesis is primarily mediated by CD8+ cytotoxic T lymphocytes via interferon-γ-driven pathways, leading to targeted destruction of melanocytes. However, recent studies have highlighted the importance of immune regulatory mechanisms, including PD-L1-expressing neutrophils and FOXP3+ regulatory T cells, which limit excessive immune-mediated damage and may contribute to the self-limited course and repigmentation observed in some lesions. Additional cytotoxic mediators, particularly granulysin, appear to strengthen melanocyte-directed cytotoxicity, while dendritic cells, macrophages, Langerhans cells, neutrophils, and natural killer cells contribute to antigen presentation, tissue remodeling, and immune resolution. HN shares key immunopathogenic features with vitiligo and melanoma regression but differs from both conditions due to its localized, tightly regulated behavior. Moreover, the halo phenomenon is not restricted to melanocytic lesions, supporting the view that it reflects a broader immune pattern rather than a disease-specific entity. This review provides a comprehensive synthesis of the clinical, histopathological, immunological, diagnostic, and translational aspects of halo nevus. Based on the available evidence, we propose a self-limited melanocyte autoimmunity model to explain the characteristic balance between melanocyte destruction, immune regulation, and spontaneous resolution observed in halo nevi.

## Simple Summary

Halo nevus is a benign melanocytic lesion characterized by a white halo surrounding a mole. Although traditionally considered an innocuous clinical finding, recent research has shown that halo nevus represents a highly organized immune response directed against melanocytes. This review summarizes current knowledge on the clinical, histopathological, and immunological features of halo nevus and highlights its close relationship with vitiligo, melanoma regression, and melanoma-associated leukoderma. Particular attention is given to the balance between immune-mediated melanocyte destruction and immune regulatory mechanisms that limit tissue damage and allow spontaneous resolution or repigmentation. We also discuss current diagnostic challenges, including distinguishing halo nevus from melanoma, and provide practical guidance for clinical management. Finally, we propose that halo nevus represents a naturally occurring model of self-limited melanocyte autoimmunity, offering new insights into pigmentary disorders, immune tolerance, and tumor immunology that may be relevant for future therapeutic strategies.

## 1. Introduction

Halo nevus (HN) is a melanocytic lesion defined by a distinctive rim of depigmentation encircling a central nevus. Also known as Sutton nevus, leukoderma acquisitum centrifugum, perinevoid vitiligo, or leukopigmentary nevus, HN most commonly occurs in children, adolescents, and young adults, with an estimated prevalence of about 1% in the general population [1,2]. The typical course of HN begins with the development of a depigmented halo, followed by gradual fading of the central nevus, which may eventually disappear, and later partial or complete repigmentation of the surrounding skin [1,3]. This sequence reflects an evolving immunological process. The central nevus and the surrounding depigmentation represent two visible aspects of a localized immune response targeting melanocytes and nevus cells. Notably, HN, vitiligo, and melanoma-associated depigmentation are all considered immunological leukodermas, linked by immune responses against melanocyte-associated antigens, though their clinical outcomes differ significantly [2,3].

Vitiligo is a chronic autoimmune disorder characterized by progressive loss of functional melanocytes and clinically evident depigmented macules and patches. Its pathogenesis involves genetic predisposition, oxidative stress, innate immune activation, adaptive cytotoxic responses, and persistent memory T-cell activity [4,5,6,7]. HN shares several of these pathways but remains localized and frequently self-limited. This difference makes HN particularly interesting because it may illustrate how melanocyte-directed autoimmunity can be contained and resolved.

The link between HN and melanoma regression further increases its relevance. Melanoma regression reflects an immune response against malignant melanocytes and may clinically or histologically resemble the halo phenomenon. Both HN and regressing melanoma may show lymphocytic infiltration and melanocyte loss, but their biological meanings are distinct: HN represents a controlled immune response against benign melanocytes, whereas melanoma regression occurs in the context of tumor immune surveillance and tumor immune escape [8,9,10].

For these reasons, HN offers an opportunity to examine the balance between immune activation and regulation. Its analysis may offer insights into autoimmune pigmentary disorders, melanoma biology, immune checkpoint regulation, and the mechanisms that determine whether melanocyte-directed immunity remains localized, becomes systemic, or fails to eliminate malignant cells.

## 2. Clinical Features and Natural History

Clinically, HN appears as a central melanocytic nevus surrounded by a symmetrical depigmented halo. The central nevus may be junctional, compound, intradermal, congenital, blue, Spitz-like, or, less commonly, another melanocytic subtype [1,2,11]. The lesions are usually located on the trunk, especially the back, although they may also occur on the face, neck, extremities, and other body sites. Multiple HNs may occur, particularly in younger patients and in individuals with associated vitiligo [1,2].

**Figure 1:**
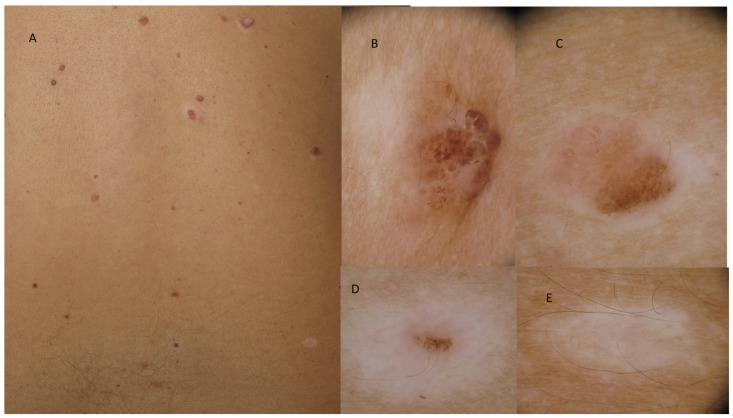
Clinical presentation and evolution of halo nevi: (**A**) Multiple halo nevi on the trunk of a 27-year-old man. (**B**,**C**) Representative halo nevi showing a well-defined rim of depigmentation surrounding a central melanocytic nevus. (**D**) Advanced-stage halo nevus with near-complete regression of the central melanocytic lesion, leaving only a small residual pigmented focus within the depigmented halo. (**E**) Late-stage lesion demonstrating complete disappearance of the nevus, with persistence of the depigmented halo as the sole clinical finding. This figure is obtained from the archive of the Dermato-Oncology Unit, Istituto Europeo di Oncologia, Milan, Italy. It is not intended to support new scientific findings and is enclosed solely for illustrative and educational purposes.

**Figure 2:**
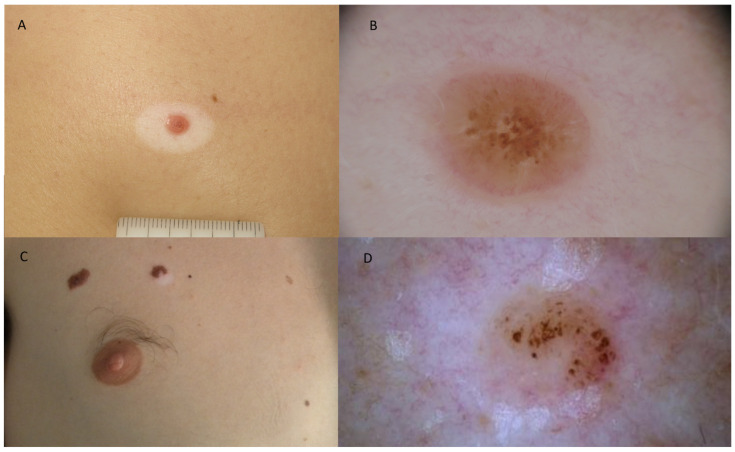
Clinical and dermoscopic appearance of a halo nevus: (**A**) Halo nevus on the trunk of a young female. A well-demarcated and symmetric rim of depigmentation surrounds the central melanocytic lesion. (**B**) Dermatoscopy of the halo nevus shown in (**A**) shows a light brown pigmented lesion with a homogeneous and globular pigmentation pattern. A faint erythematous ring is visible at the periphery of the nevus. The surrounding whitish, structureless background represents the depigmented halo (magnification ×10). (**C**) Halo nevus on the chest of a young man. A well-circumscribed, asymmetrical, depigmented halo surrounds a central melanocytic lesion. (**D**) Dermatoscopy demonstrates a melanocytic nevus with a globular pigmentation pattern. The surrounding structureless white area corresponds to the halo of depigmentation (magnification ×10). This figure is obtained from the archive of the Dermato-Oncology Unit, Istituto Europeo di Oncologia, Milan, Italy. It is not intended to support new scientific findings and is enclosed solely for illustrative and educational purposes.

The clinical evolution of HN has traditionally been divided into stages. The first stage consists of the development of a depigmented halo around a central nevus. The second stage involves progressive fading of the central nevus. In the third stage, the central nevus may disappear completely, leaving only a depigmented macule. In the fourth stage, repigmentation may occur over months or years [3]. However, this sequence is variable. Some lesions persist for long periods, some repigment incompletely, and others may remain clinically stable (Figure 1 and Figure 2).

A retrospective series on the natural history of HN described spontaneous regression of the central nevus in many patients, although the timing and completeness of regression varied [3]. The surrounding depigmented halo may persist even after the central lesion disappears, thus suggesting that melanocyte loss in the halo might reflect a broader local immune response.

Halo formation may also occur around congenital melanocytic nevi, although this is less common than around acquired nevi. Congenital HN might also become targets of immune-mediated regression, particularly in childhood [11,12]. This observation broadens the clinical spectrum of HN and supports the idea that the immune response is directed against melanocytic antigens rather than exclusively against acquired nevus cells.

Rare variants such as halo Spitz nevi and polypoid Spitz nevi with a halo reaction further complicate the clinical and histopathological spectrum. Spitz nevi can occasionally develop a halo reaction, and inflammation may obscure some of the usual criteria used to distinguish Spitz nevus from melanoma. In such cases, architectural symmetry, maturation, mitotic pattern, p16 expression, BAP1 status, and molecular findings may help support a benign diagnosis [13].

HN in children and adolescents is usually benign and may be managed conservatively when it shows typical morphology. In contrast, halo-like depigmentation around a lesion in adulthood, particularly after age 40, warrants careful assessment because melanoma with regression may occasionally mimic an HN [2,9]. Thus, the diagnostic meaning of a halo depends strongly on age, morphology, dermoscopic features, evolution, and clinicopathological correlation.

## 3. Histopathological Features of Halo Nevus

**Figure 3:**
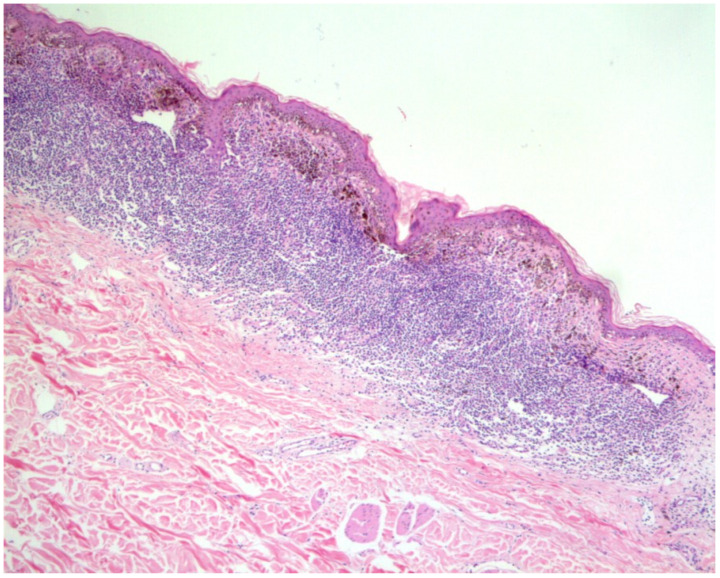
Histopathology of a halo nevus shows a compound melanocytic nevus with preserved architectural symmetry and bland cytologic features. A dense dermal lymphocytic inflammatory infiltrate partially obscures the intradermal nevus component. Numerous dermal melanophages are present (H&E, magnification ×20). This figure is obtained from the archive of the Dermato-Oncology Unit, Istituto Europeo di Oncologia, Milan, Italy. It is not intended to support new scientific findings and is enclosed solely for illustrative and educational purposes.

Histologically, HN is characterized by a dense inflammatory infiltrate surrounding and infiltrating nevus nests, with progressive destruction of nevus cells and melanocytes. The infiltrate is predominantly lymphocytic and often obscures the melanocytic component. In more advanced stages, nevus cells may become sparse or absent, leaving fibrosis, melanophages, and residual inflammatory changes [2,9] (Figure 3).

The histopathological evolution of HN can be divided into stages. In the pre-regression stage, the melanocytic nevus remains largely preserved, with early accumulation of antigen-presenting cells such as Langerhans cells. During early regression, nevus nests become blurred and are infiltrated by lymphocytes and dendritic cells. In late regression, nevus cells are sparse and poorly defined, while a dense band-like inflammatory infiltrate, predominantly composed of CD8+ T cells, macrophages, and antigen-presenting cells, surrounds and replaces the melanocytic component. In complete regression, nevus cells disappear, and the inflammatory infiltrate decreases markedly [3]. This staged model shows that clinical depigmentation and histological inflammation do not always evolve synchronously.

**Figure 4:**
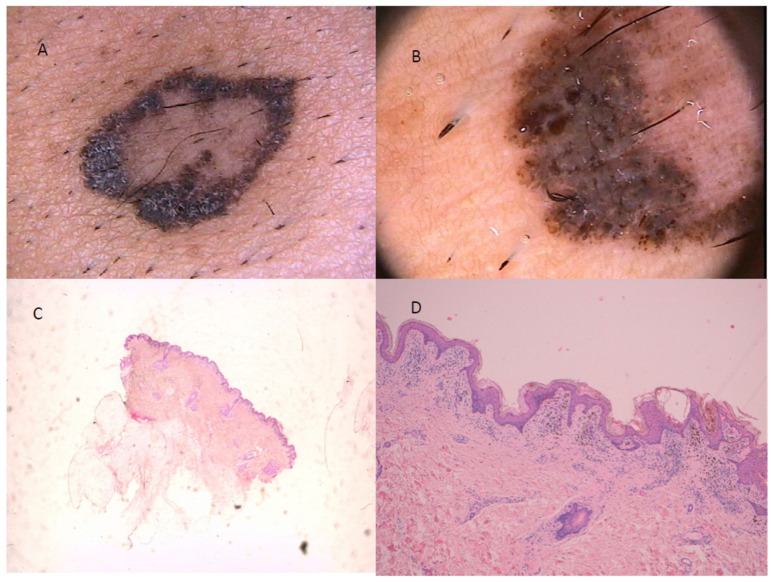
Clinical, dermatoscopic, and histologic features of an extensively regressing melanocytic lesion: (**A**) Clinical image showing a roughly circular melanocytic lesion undergoing regression. The lesion displays a central depigmented area with residual pigmentation persisting at the periphery. (**B**) Dermoscopic examination reveals a pigmented area composed of globules and dots surrounding a central structureless depigmented area, corresponding to extensive regression (magnification ×10). (**C**) Histopathological examination demonstrates marked regression changes, including dermal fibrosis, neovascularization, and a chronic lymphocytic inflammatory infiltrate. Numerous dermal melanophages are present (H&E, magnification ×5). (**D**) Asymmetrical epidermal alterations are evident, with hyperplastic rete ridges in the pigmented portion of the lesion and focal epidermal atrophy in the regressed area (H&E, magnification ×10). This figure is obtained from the archive of the Dermato-Oncology Unit, Istituto Europeo di Oncologia, Milan, Italy. It is not intended to support new scientific findings and is enclosed solely for illustrative and educational purposes.

The most relevant histopathological challenge is distinguishing an HN from melanoma with regression. Both may show lymphocytic inflammation, melanocyte loss, fibrosis, and melanophages. However, HN usually maintains symmetry, maturation of melanocytes with depth, and limited cytological atypia. Melanoma, by contrast, typically shows asymmetry, architectural disorder, lack of maturation, pagetoid spread, and cytological atypia extending throughout the lesion [9,14] (Figure 4).

Immunohistochemistry may support the diagnosis but should not be interpreted in isolation. Markers such as HMB-45, Melan-A, SOX10, Ki-67, CD3, CD4, CD8, FOXP3, CD25, and PD-1 can help characterize melanocytic maturation and the immune infiltrate [8,9,10]. Comparative studies show that HN often exhibits a CD8-predominant immune infiltrate, whereas regressing melanoma may show relatively higher CD4/CD8 ratios and different regulatory profiles [8,9]. Recent immunophenotypic data demonstrated that atypical Sutton nevi had higher CD8/CD3 ratios and higher CD25/CD4, FOXP3/CD4, and PD1/CD4 ratios compared with melanomas with regression, suggesting a distinctive cytotoxic-regulatory immune environment in HN [9]. This distinction supports the interpretation of HN as an immune response that is not merely destructive but actively regulated.

In inflamed or regressing melanocytic lesions arising during immunotherapy, additional immunohistochemical markers, including PRAME, p16, SOX10, Melan-A, HMB-45, and Ki-67, may help distinguish benign inflamed nevi from melanoma. However, interpretation must always integrate morphology, clinical context, and lesion evolution [15].

## 4. Immunopathogenesis of Halo Nevus

The immunopathogenesis of HN is primarily mediated by CD8+ T cells, which are the dominant effector cells within the inflammatory infiltrate and are believed to recognize melanocyte-associated antigens expressed by nevus cells and surrounding melanocytes [2,4,8]. Once activated, CD8+ T cells induce melanocyte apoptosis through different cytotoxic pathways.

Interferon-γ is a central amplifier of this immune response. It increases antigen presentation by upregulating MHC class I molecules and promotes the recruitment of additional effector T cells via chemokines such as CXCL9 and CXCL10 [3,4,5,6,7]. These pathways are also central in vitiligo and support the concept that HN and vitiligo share common melanocyte-associated autoantigens, thus raising the question of whether these conditions also share them.

Although the precise initiating autoantigen(s) responsible for triggering HN remain unknown, evidence suggests that the immune response is directed against melanocyte differentiation antigens shared by normal melanocytes, nevus cells, and melanoma cells [3,4,5,6,7,8,16,17,18,19]. Among the candidates are MART-1 (Melan-A), gp100, tyrosinase, tyrosinase-related protein-1 (TRP-1), and tyrosinase-related protein-2 (TRP-2), all of which are recognized by autoreactive CD8+ T lymphocytes in vitiligo and by tumor-reactive T cells in melanoma [4,5,6,7,8,16,17,20,21]. Their expression in benign melanocytic nevi suggests that they also represent relevant immune targets in HN [2,3,16,17]. Rather than being directed against a unique HN-specific antigen, the available evidence supports the hypothesis that HN develops through recognition of a shared repertoire of melanocyte differentiation antigens. This antigenic overlap provides a mechanistic explanation for the close immunological relationship among HN-, vitiligo-, and melanoma-associated immune responses [3,4,5,6,7,8,16,17,22,23]. However, unlike vitiligo, in which several melanocyte autoantigens have been extensively characterized [4,5,6,7,20,21], the hierarchy of antigen recognition and the initiating autoantigen(s) responsible for triggering the immune response in HN remain incompletely understood. Future studies combining antigen-specific T-cell analyses, T-cell receptor repertoire sequencing, and spatial transcriptomics may help clarify the antigenic determinants driving this localized autoimmune response [17,24,25].

An additional mechanism that may contribute to the evolution of the immune response in HN is epitope spreading, a phenomenon whereby an immune response initially directed against a restricted set of antigens progressively broadens to include additional epitopes released during tissue injury. Epitope spreading has been implicated in several autoimmune diseases, including vitiligo, where progressive melanocyte destruction may expose previously concealed melanocyte-associated antigens, thereby amplifying autoreactive T-cell responses [4,5,6,7].

Although direct experimental evidence supporting epitope spreading in HN is currently lacking, this mechanism represents a biologically plausible hypothesis. The initial immune response may be directed against melanocyte differentiation antigens expressed by nevus cells. Progressive immune-mediated destruction of nevus cells could subsequently release additional melanocyte-associated antigens that are captured and processed by dendritic cells, leading to activation of autoreactive CD8+ T cells directed against surrounding normal melanocytes [3,16,17,24].

This hypothesis may explain the progressive enlargement of the depigmented halo during lesion evolution. It may also provide a mechanistic explanation for the occasional progression from localized HN to generalized vitiligo in genetically predisposed individuals [26,27,28]. Nevertheless, unlike vitiligo, HN remains spatially restricted, suggesting that effective local immune regulatory mechanisms limit further antigen spreading and prevent progression toward chronic autoimmunity [29,30].

Comparative transcriptomic analyses between HN, vitiligo, normal nevi, and melanoma have identified overlapping interferon-driven, cytotoxic T-cell-associated, and antigen-processing signatures [16,17].

Dendritic cells are essential for initiating this immune response. They process melanocyte-derived antigens and present them to naïve T cells, promoting differentiation toward cytotoxic effector phenotypes. Langerhans cells and other dendritic cell populations are evident in early histological stages, supporting their role in antigen presentation and immune priming [3,24]. In addition, abnormal melanocyte metabolism and oxidative stress may provide danger signals that activate dendritic cells. Metabolic changes, such as UDP–glucose accumulation, may serve as danger-associated molecular patterns and contribute to innate immune activation [24,31].

In HN, macrophages contribute to phagocytosis of melanocyte and nevus cell remnants, antigen presentation, cytokine production, tissue remodeling, and pigmentary changes. CD68+ macrophages are evident in regressing lesions, and macrophage involvement increases during stages of nevus destruction [32,33]. More recent literature suggests that macrophages influence pigmentation through both phagocytosis and secretion of cytokines such as IFN-γ, TNF, GM-CSF, IL-1, IL-6, IL-8, and IL-18 [34]. In HN, macrophages may therefore be involved not only in clearing cellular debris but also in shaping the local inflammatory and pigmentary microenvironment.

Natural killer cells also contribute to the early immune response through cytotoxicity and cytokine production. Although they appear less abundant than CD8+ T cells, natural killer cells expressing cytotoxic mediators such as granulysin have been described in HN lesions, suggesting a supportive role in the destruction of melanocytic cells [3,22,23,35].

Neutrophils are among the most recent additions to the immunopathogenic model of HN. PD-L1-expressing neutrophils have been identified in HN, where they interact with CD8+ T cells and suppress their cytotoxic function. Zhang et al. demonstrated that interferon-γ can induce PD-L1 expression in neutrophils and that these PD-L1+ neutrophils suppress CD8+ T-cell function, promote CD8+ T-cell apoptosis, and may contribute to leukoderma repigmentation [29]. These findings indicate that PD-L1-expressing neutrophils may contribute to local regulation of CD8+ T-cell activity and immune resolution [29].

FOXP3+ regulatory T cells are increased in the early stages of HN, and the FOXP3+/CD8+ T-cell ratio is higher in early HN lesions [30]. This suggests that immune suppression is not merely a late consequence of regression but may be present from the beginning of lesion development. Taken together, these findings indicate that HN is characterized by a complex immune microenvironment in which cytotoxic effector cells coexist with multiple regulatory immune populations. The coordinated interaction among these cell types is likely to influence both melanocyte destruction and subsequent immune resolution [29,30,32,33,34,35].

## 5. Halo Nevus and Vitiligo: A Shared Autoimmune Spectrum

The relationship between HN and vitiligo is one of the crucial aspects of this topic. Both conditions involve immune-mediated melanocyte destruction, CD8+ T-cell activation, interferon-γ signaling, and chemokine-mediated recruitment of effector T cells [4,5,6,7,16,17,29]. Clinically, HN may precede, coexist with, or follow vitiligo, and patients with multiple HN appear more likely to develop vitiligo than those with isolated lesions [26,27,28].

Van Geel et al. emphasized that HN may be associated with the development or presence of vitiligo in selected patients [26]. Zhou et al. investigated factors associated with the development of vitiligo in patients with HN and reinforced the clinical link between these entities [27]. Case reports of segmental vitiligo arising from HN further support the idea that localized immune activation may expand into broader depigmentation patterns in predisposed individuals [28].

Despite these similarities, HN and vitiligo differ in clinical behavior. Vitiligo is usually chronic, may be progressive, and often involves distant areas of the skin, while HN is usually localized and may resolve spontaneously. The difference likely depends on the strength and persistence of regulatory mechanisms. In HN, PD-L1+ neutrophils and FOXP3+ T-regs may restrain cytotoxic activity; in vitiligo, regulatory failure, resident memory T cells, oxidative stress, macrophage-associated cytokine loops, and chronic interferon-driven pathways may maintain disease activity [4,5,6,7,29,30,34].

Vitiligo is well recognized as a systemic autoimmune disorder associated with several autoimmune comorbidities, particularly autoimmune thyroid disease [36,37,38,39,40,41,42]. Large cohort studies and meta-analyses support these associations and justify clinical awareness of thyroid dysfunction in patients with vitiligo.

## 6. Thyroid Disease, Hypothyroidism, and Autoimmune Associations

Meta-analytic data summarized in recent studies indicate substantially increased odds of autoimmune thyroiditis and hypothyroidism among patients with vitiligo, supporting the recommendation that thyroid dysfunction should be considered in the clinical evaluation of patients with vitiligo, especially when symptoms, family history, or extensive disease is present [37,38].

The relationship between HN and thyroid disease, when present, is likely indirect and mediated by concomitant vitiligo or by a broader autoimmune predisposition rather than by HN itself. Current evidence does not support routine thyroid or autoimmune laboratory screening in patients with isolated HN. Evaluation should be individualized and considered mainly in patients with multiple HNs, concomitant vitiligo, suggestive clinical symptoms, or a personal or family history of autoimmune disease [36,37,38,39,40,41,42].

Genetic and immunological overlap between vitiligo and other autoimmune diseases may provide a biological explanation for autoimmune clustering, but direct evidence for such clustering in isolated HN remains insufficient [20,21].

## 7. Regression Dynamics, Repigmentation, and Immune Resolution

The regression of HN is a visible example of immune-mediated tissue remodeling. The central nevus may gradually disappear as cytotoxic lymphocytes destroy nevus cells. At the same time, the surrounding depigmented halo reflects loss or dysfunction of normal melanocytes. The later stage of repigmentation suggests that immune attack can cease or decrease sufficiently to allow melanocyte recovery or migration from hair follicles and surrounding skin [1,3,29].

The biological mechanisms underlying repigmentation have been extensively investigated in vitiligo and provide a useful framework for understanding the spontaneous repigmentation observed in HN [4,5,6,7]. In vitiligo, repigmentation typically begins in a perifollicular pattern. It is primarily driven by melanocyte stem cells residing within the hair follicle bulge, which constitutes the principal stem-cell niche for the melanocyte lineage in adult skin [18]. Following adequate suppression of the autoimmune response, these stem cells become activated, proliferate, migrate centrifugally into the depigmented epidermis, and differentiate into mature melanocytes, progressively restoring pigmentation [4,5,6,7,18]. Successful repigmentation requires not only activation of melanocyte stem cells but also restoration of a permissive cutaneous immune microenvironment through attenuation of IFN-γ-driven inflammation, reduced CXCL9/CXCL10-mediated recruitment of cytotoxic T cells, and decreased activity of resident memory T cells, all of which otherwise impair melanocyte regeneration [4,5,6,7].

Whether identical regenerative mechanisms operate in HN remains unknown. Nevertheless, the spontaneous and often complete repigmentation observed in many lesions strongly suggests that follicular melanocyte stem cells similarly contribute to the repopulation of the depigmented halo once local immune-mediated melanocyte destruction has resolved [3,16,17,29,43]. Beyond activation of melanocyte stem cells, restoration of local immune tolerance appears to be essential for effective melanocyte regeneration. Recent evidence suggests that PD-L1-expressing neutrophils, together with FOXP3+ regulatory T cells, contribute to this process by dampening local cytotoxic immune responses and creating a permissive microenvironment for melanocyte regeneration [29,30]. Zhang et al. demonstrated that interferon-γ induces PD-L1 expression in neutrophils, which in turn suppress CD8+ T-cell function and promote apoptosis of activated T cells, providing a mechanistic explanation for the self-limited behavior of HN and its capacity for spontaneous repigmentation [29]. The localized and self-limited nature of the inflammatory response in HN may therefore create a more favorable regenerative niche than that observed in vitiligo, where persistent resident memory T cells and chronic IFN-γ signaling frequently sustain disease activity [4,5,6,7].

Other immune cell populations may also participate in repigmentation. In some cases of HN after excision, differences in the immune microenvironment have been reported between improved and non-improved cases, suggesting that the balance among CD8+ cells, macrophages, B cells, dendritic cells, and PD-L1+ cells may influence whether depigmentation persists or improves [43]. This supports the concept that repigmentation represents a regulated immunological and regenerative process. In vitiligo, therapeutic efforts increasingly aim to suppress interferon-γ signaling, inhibit JAK pathways, reduce pathogenic T-cell activity, and promote melanocyte regeneration [4,5,6,7]. HN may therefore serve as a naturally occurring model of immune resolution, allowing investigators to compare why localized immune responses resolve in some contexts but persist in others.

## 8. Halo Phenomenon Beyond Melanocytic Lesions

The halo phenomenon is not limited to melanocytic nevi. Depigmented halos have been reported around non-melanocytic lesions, including angiokeratomas and seborrheic keratoses [44,45,46]. These reports challenge the assumption that a halo necessarily indicates a melanocyte-specific process. Instead, halo formation may represent a localized immune pattern that can occur around different antigenic targets.

Halo angiokeratoma has been described as a rare example of depigmentation surrounding a vascular lesion, suggesting that local immune activation may secondarily affect melanocytes in the surrounding skin [44]. Halo seborrheic keratosis similarly supports the view that immune-mediated depigmentation can occur around non-melanocytic lesions [45]. The Meyerson phenomenon and other inflammatory reactions around seborrheic keratoses further reinforce the concept that perilesional immune activation can produce clinical patterns that resemble HN without being primarily melanocytic in origin [46].

This has diagnostic implications. A halo should not be considered diagnostic of a benign melanocytic lesion by itself. Rather, it should be understood as a morphological sign of immune activity. The underlying lesion may be benign, inflammatory, melanocytic, non-melanocytic, or rarely malignant.

## 9. Halo Nevus, Melanoma Regression, and Tumor Immunology

The relationship between HN and melanoma regression is central to the translational relevance of this topic. Melanoma can undergo partial or complete regression due to host immune responses directed against tumor antigens. This process may involve CD8+ T cells, macrophages, antigen-presenting cells, cytokines, and immune checkpoints [8,9,10,47]. Clinically and histologically, regressing melanoma may resemble HN because both conditions can show melanocyte loss and lymphocytic inflammation.

However, the two conditions differ fundamentally. HN usually represents effective immune-mediated destruction of benign melanocytes within a regulated environment. Melanoma regression occurs in a malignant context in which immune destruction may be incomplete, and tumor immune escape may occur. Some melanoma clones may evade immune surveillance through antigen loss, reduced antigen presentation, altered interferon signaling, recruitment of suppressive cells, or checkpoint-mediated inhibition [8,9,10,19,47].

Comparative immunophenotyping has provided useful distinctions. HN often shows a CD8-predominant infiltrate and increased expression of regulatory markers such as PD-1, FOXP3, and CD25, consistent with a robust yet regulated immune response [8,9,10]. In contrast, melanoma regression may show different CD4/CD8 ratios and less effective compensatory regulation. Brugués et al. reported significantly higher CD8/CD3 ratios in HN than in regressing melanomas, whereas regressing melanomas showed higher CD4/CD3 and CD4/CD8 ratios; they also found higher CD25/CD4, FOXP3/CD4, and PD1/CD4 ratios in HN [9]. These findings support the idea that HN is characterized by coordinated cytotoxicity and regulation.

Vitiligo and melanoma also have an epidemiological relationship. Several studies have reported a reduced risk of melanoma and keratinocyte cancers in patients with vitiligo, although results vary by population, ethnicity, study design, ultraviolet exposure, and surveillance patterns [48,49,50,51,52]. Delcoigne et al. conducted a large Swedish matched-cohort study. They reported approximately 50% lower risk of melanoma in individuals with vitiligo and autoimmune alopecia compared with matched controls, with no reduction in non-cutaneous solid or hematological cancers [48]. These findings suggest that melanocyte-directed immune responses may contribute to protection against skin cancer, although behavioral factors such as sun avoidance may also play a role.

The phenomenon of melanoma-associated vitiligo and vitiligo-like depigmentation during immune checkpoint inhibitor therapy further supports the connection between melanocyte autoimmunity and antitumor immunity [19,53,54,55,56]. In melanoma patients treated with immune checkpoint inhibitors, depigmentation can reflect immune activation against shared melanocytic antigens and is often associated with favorable tumor responses [19,53,54,55,56]. Immune checkpoint inhibitors may also induce inflammation or regression in benign nevi, sometimes without a clinically evident halo, creating diagnostic challenges. Careful morphological assessment along with selected markers such as PRAME, p16, SOX10, Melan-A, and Ki-67 may help distinguish benign immune-mediated changes from melanoma [15]. This duality suggests that the same immune response that causes autoimmunity against normal melanocytes may contribute to tumor control when directed against malignant melanocytes.

## 10. Immune Checkpoints, Immunotherapy, and Therapeutic Implications

The PD-1/PD-L1 pathway is a critical regulator of T-cell activation and peripheral tolerance. In HN, PD-L1+ neutrophils may act as local regulators, suppressing CD8+ T-cell activity and promoting lesion resolution [29]. In melanoma therapy, PD-1 blockade removes inhibitory signaling and enhances antitumor cytotoxicity but may also trigger melanocyte destruction and vitiligo-like depigmentation [53,54,55,56].

This creates an apparent paradox. In HN, PD-L1 signaling may help resolve inflammation and prevent chronic autoimmunity. In melanoma, PD-L1 expression may contribute to tumor immune escape. The biological effect of PD-1/PD-L1 signaling therefore depends on context. In a benign immune reaction, checkpoint activation may be protective; in cancer, it may be exploited by tumor cells. Understanding this context dependence may help guide future therapies. If PD-L1+ neutrophils promote repigmentation in HN and vitiligo, strategies that enhance local regulatory pathways could theoretically reduce melanocyte destruction. Conversely, in melanoma, checkpoint blockade is used to intensify cytotoxic responses. HN thus provides a natural example of checkpoint-mediated immune balance and may help clarify how to restore immune balance in autoimmune disease while preserving antitumor immunity.

## 11. Triggers, Trauma, Laser Therapy, and Iatrogenic Modulation

The development or exacerbation of HN may be influenced by environmental, mechanical, and iatrogenic factors. Physical trauma, chronic rubbing, inflammatory injury, laser therapy, and immune-modulating drugs may expose melanocyte antigens or induce local danger signals that activate immune responses. This is relevant because HN is not only a spontaneous event but may also arise or evolve in response to external stimulation.

A notable example is the development of double HNs and vitiligo following laser therapy. In this setting, incomplete destruction of a melanocytic lesion may leave residual melanocytic cells that continue to act as antigenic targets, potentially amplifying local and systemic melanocyte-directed autoimmunity [57]. This supports caution when considering destructive procedures for melanocytic nevi, particularly in patients with a personal or family history of vitiligo or autoimmune disease.

Trauma-induced antigen exposure and Koebner-like mechanisms may also explain why HN and vitiligo occasionally emerge after local injury. These observations suggest that external triggers may convert a previously tolerated melanocytic lesion into an immune target in predisposed individuals.

## 12. Genetic, Molecular, and Bioinformatic Insights

Genetic and molecular studies of vitiligo provide additional context for HN. Vitiligo is associated with variants in immune regulation, antigen presentation, melanocyte biology, oxidative stress, apoptosis, and pigmentation pathways [20,21,58]. Some loci show inverse relationships with melanoma risk, supporting the idea that genetic predisposition to melanocyte autoimmunity may enhance immune surveillance against melanoma [20,21,48,49,50,51,52].

Recent work on apoptosis-related polymorphisms suggests that intrinsic melanocyte vulnerability may also contribute to disease. Daneshparvar et al. investigated the BAX-248G>A and BCL2-938C>A polymorphisms in vitiligo and found associations between vitiligo risk and variants that affect apoptosis regulation [58]. Although this study focuses on vitiligo rather than HN, it is relevant because melanocyte apoptosis is a shared final pathway in pigmentary autoimmunity.

Bioinformatic analyses further demonstrate that vitiligo involves dysregulation of melanogenesis-related genes and immune cell infiltration. Ma and Wang identified differentially expressed melanogenesis-related genes, including CALM2, KIT, and OCA2, and reported downregulation of melanogenesis pathways, along with altered immune infiltration, particularly involving T helper and Th2 cells [25]. These findings suggest that melanocyte dysfunction and immune activation are interconnected rather than independent processes.

Macrophages participate in pigmentary disorders by phagocytosing melanin and secreting cytokines that can promote or inhibit melanogenesis [34]. In vitiligo, macrophage-associated inflammatory pathways, macrophage migration inhibitory factor, altered M1/M2 balance, and cytokines such as IL-6, TNF, and IFN-γ may contribute to melanocyte dysfunction and immune amplification [34]. These mechanisms may not fully overlap with HN, but they are relevant because macrophages and melanophages are consistently present during nevus regression and pigmentary remodeling.

Such data are important for HN because the central biological event is also the destruction of melanocytes in an immune-rich microenvironment. Future studies comparing HN, vitiligo, regressing melanoma, and stable nevi using single-cell transcriptomics, spatial transcriptomics, multiplex immunofluorescence, and T-cell receptor sequencing could clarify how similar immune pathways lead to different outcomes.

## 13. Diagnostic Challenges and Clinical Management

The distinction between HN, melanoma-associated leukoderma, and regressing melanoma relies on the integration of clinical history, patient age, lesion morphology, dermoscopic findings, and, when required, histopathology and immunohistochemistry.

From a histopathological perspective, HN, melanoma-associated leukoderma, regressing melanoma, and immunotherapy-induced vitiligo-like reactions share several features, including melanocyte loss, pigment incontinence, melanophages, and variable lymphocytic inflammation [8,9,10,19,32,33,53,54,55,56]. Nevertheless, distinguishing characteristics are usually present. HN is characterized by a dense, predominantly CD8+ lymphocytic infiltrate surrounding a cytologically bland and architecturally symmetrical melanocytic nevus, frequently associated with melanophages but generally lacking significant fibrosis or prominent interface dermatitis [8,9,10,32,33]. In contrast, regressing melanoma usually demonstrates residual atypical melanocytic proliferation, architectural asymmetry, lack of maturation, pagetoid spread, variable fibrosis, and regression-associated vascular changes [8,9,10,19,47]. Melanoma-associated leukoderma typically involves clinically normal depigmented skin and is characterized by loss of epidermal melanocytes with only sparse superficial lymphocytic infiltrates, without an associated melanocytic proliferation [19,53,54,55,56]. Similarly, immunotherapy-induced vitiligo-like reactions usually show complete or near-complete epidermal melanocyte loss, mild interface dermatitis, and superficial perivascular lymphocytic infiltrates but lack an underlying melanocytic lesion [15,53,54,55,56] (Table 1). Therefore, careful correlation of histopathological findings with clinical presentation, dermoscopy, and immunohistochemistry remains essential for accurate diagnosis [8,9,10,15,19].

Young patients presenting with a symmetric halo surrounding a long-standing melanocytic nevus are more likely to have an HN. Conversely, new-onset halo lesions in adults, particularly when associated with asymmetry, irregular pigmentation, rapid evolution, or a personal history of melanoma, should prompt excision and histopathological examination. Immunohistochemical markers, including PRAME, SOX10, Melan-A, HMB-45, and Ki-67, may provide additional diagnostic support in selected cases, although morphology remains the diagnostic gold standard [8,9,10,15].

The main clinical challenge is distinguishing typical HN from melanoma with regression. In children and adolescents, a symmetric halo around a benign-appearing nevus can often be monitored clinically and dermoscopically. In adults, especially older adults, new halo formation around a pigmented lesion should be evaluated with caution [2,8,9,10,14].

**Figure 5:**
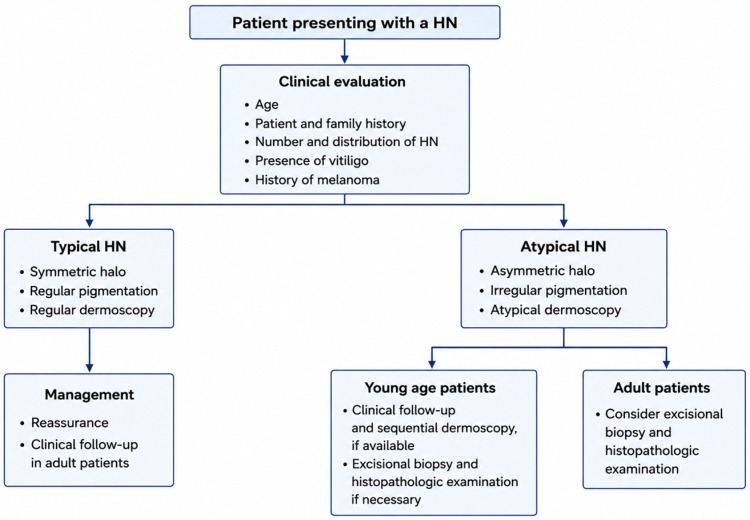
Proposed diagnostic and clinical management algorithm for patients presenting with a halo nevus (HN). Initial evaluation should include patient age, clinical history, number and distribution of halo nevi, personal or family history of autoimmune disease, presence of vitiligo, and previous melanoma. Typical halo nevi, characterized by a symmetric depigmented halo, regular pigmentation, and a typical dermoscopic pattern, can generally be managed conservatively with reassurance and clinical and/or dermoscopic follow-up. In contrast, atypical halo lesions, particularly those occurring in adults or showing asymmetry, irregular pigmentation, or atypical dermoscopic features, require a more cautious approach. In young patients, short-term clinical and dermoscopic monitoring may be considered when clinical suspicion is low, whereas excisional biopsy with histopathological examination should be performed if diagnostic uncertainty persists. In adults, atypical halo lesions should generally undergo excisional biopsy to exclude regressing melanoma. The algorithm emphasizes individualized management based on clinical context, age, lesion morphology, and dermoscopic findings.

Features that favor HN include young age, symmetry, regular borders, uniform halo, absence of atypical dermoscopic features, stability or gradual regression, and absence of alarming clinical changes. Features that raise concern include adult-onset, asymmetry, irregular pigmentation, atypical dermoscopic features (atypical pigmented network, blue-white veil, or ulceration), rapid change, nodularity, bleeding, or a personal history of melanoma [2,8,9,10,14]. Reflectance confocal microscopy may assist in selected cases, but inflammatory cells can mimic atypical melanocytes, creating diagnostic pitfalls [59]. Sequential dermoscopy may be useful in children with typical lesions. Biopsy is appropriate in atypical lesions and when melanoma cannot be confidently excluded [14,59,60] (Figure 5).

The clinician should evaluate the patient as a whole, particularly when HNs are multiple or associated with extralesional depigmented patches.

## 14. Halo Nevus as a Natural Model of Immune Resolution

**Figure 6:**
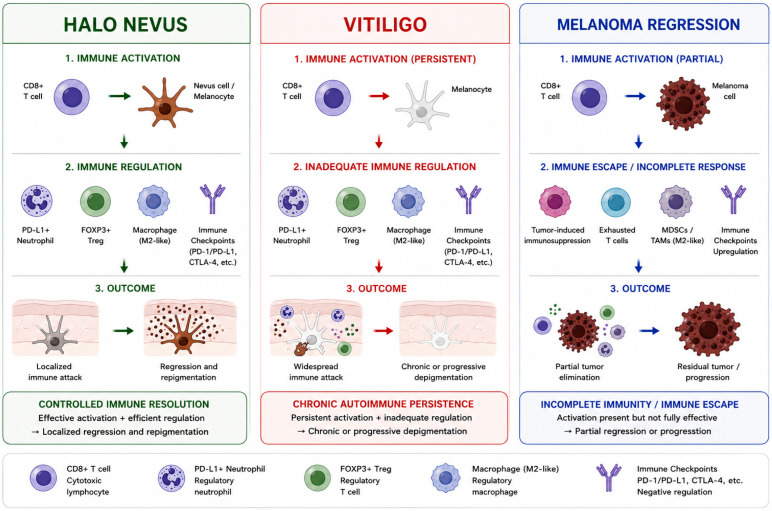
The self-limited melanocyte autoimmunity model. This schematic compares the main outcomes of melanocyte-directed immune responses in halo nevus, vitiligo, and melanoma regression. In halo nevus, cytotoxic immune activation is effectively balanced by regulatory mechanisms, including PD-L1–expressing neutrophils, FOXP3+ regulatory T cells, M2-like macrophages, and immune checkpoint pathways. This coordinated response limits tissue damage, promotes resolution of inflammation, and may allow lesion regression and subsequent repigmentation. In vitiligo, similar melanocyte-targeting immune mechanisms persist because regulatory control is inadequate, resulting in sustained melanocyte loss and chronic or progressive depigmentation. In melanoma regression, antitumor immune activation is present but remains incomplete because of tumor-induced immunosuppression, T-cell exhaustion, suppressive myeloid populations, and checkpoint upregulation, leading to partial tumor elimination, residual disease, or progression.

Based on the available clinical, histopathological, and immunological evidence, we propose that HN may represent a naturally occurring model of self-limited melanocyte autoimmunity. In this framework, cytotoxic immune activation is sufficiently strong to induce melanocyte and nevus cell destruction, yet sufficiently regulated to prevent chronic depigmentation, uncontrolled tissue damage, or systemic autoimmune progression. The central element of this model is the coexistence of potent cytotoxic mechanisms and active regulatory pathways. CD8+ cytotoxic T lymphocytes, supported by interferon-γ signaling, CXCL9/CXCL10-mediated recruitment, and granulysin expression, drive melanocyte destruction [4,5,6,7,16,17,22,23,61]. At the same time, FOXP3+ regulatory T cells, PD-L1-expressing neutrophils, macrophages, and immune checkpoint pathways limit excessive immune activation and promote resolution [29,30,32,33,34,35]. The result is a self-limited, local immune response (Figure 6).

This framework allows HN to be distinguished from both vitiligo and melanoma regression. In vitiligo, melanocyte-directed immunity is effective but insufficiently controlled, leading to chronic, recurrent, or progressive depigmentation [4,5,6,7,36,37,38,39,40,41,42]. In melanoma regression, immune activation may be present but is often incomplete or counteracted by tumor immune escape mechanisms [8,9,10,47,53,54,55,56]. HN, by contrast, represents a state in which melanocyte-directed immunity is both effective and regulated [3,9,29,30]. The ability of HN to undergo spontaneous stabilization and, in some cases, repigmentation suggests that endogenous pathways capable of restoring immune homeostasis remain intact and functionally effective [1,3,29,43].

Understanding the mechanisms that permit immune resolution in HN may provide insights applicable to vitiligo, immune-related adverse events during checkpoint inhibitor therapy, and other organ-specific autoimmune diseases [5,6,7,29,53,54,55,56].

## 15. Discussion

The present synthesis reinforces the concept that HN represents a localized yet highly coordinated immune response against melanocytes, sharing key immunopathogenic features with vitiligo [3,16,17,29,30]. Rather than a purely destructive phenomenon, HN represents a dynamic equilibrium between cytotoxic and regulatory immune mechanisms, in which immune activation is both effective and tightly controlled.

At the core of this process lies a CD8+ T-cell-mediated immune response against melanocyte-associated antigens. This cytotoxic pathway represents the final common mechanism of melanocyte destruction in both HN and vitiligo [4,5,6,7,22,23,61]. Similar immune activation has also been described in melanoma regression [8,9,10,19,47].

Vitiligo, melanoma-associated leukoderma, and melanoma regression share a common repertoire of melanocyte differentiation antigens, including MART-1 (Melan-A), gp100 (PMEL), tyrosinase, TRP-1, and TRP-2 [3,4,5,6,7,8,16,17,18,19]. Their distinct clinical phenotypes therefore appear to depend less on antigen specificity than on differences in the local immune context, including the balance between cytotoxic and regulatory immune mechanisms, the tissue microenvironment, and the persistence or resolution of inflammation [19,29,30]. These observations support the concept that these disorders represent distinct biological outcomes of a shared melanocyte-directed immune response [19]. A defining feature of HN, however, is the presence of effective immune regulatory mechanisms that prevent progression toward chronic autoimmunity [9,29,30].

These observations support our proposed self-limited melanocyte autoimmunity model, whereby HN represents a naturally occurring example of effective, yet self-limited, melanocyte-directed immunity [3,22,29,30].

In contrast, vitiligo represents a state of chronic autoimmune persistence, in which similar cytotoxic mechanisms operate but regulatory control is insufficient, leading to sustained melanocyte loss and disease propagation [4,5,6,7,34,36,37,38,39,40,41,42,61].

Melanoma regression represents a different scenario, characterized by incomplete or dysregulated antitumor immunity, often limited by immune escape mechanisms such as antigen loss, impaired antigen presentation, checkpoint signaling, and recruitment of suppressive immune populations [8,9,10,47,53,54,55,56].

From a translational perspective, HN may also be interpreted as a physiological counterpart of immune checkpoint modulation. The coexistence of cytotoxic activation and checkpoint-mediated regulation resembles the balance sought in cancer immunotherapy, where excessive activation leads to immune-related adverse events and insufficient activation results in treatment failure [29,53,54,55,56]. Interestingly, melanocyte-directed immunity may confer protective effects against malignancy. Epidemiological studies have reported a reduced incidence of melanoma and non-melanoma skin cancers among patients with vitiligo, suggesting that enhanced immune surveillance against melanocytic antigens may inhibit tumor development [48,49,50,51,52]. The occurrence of vitiligo-like depigmentation during immune checkpoint inhibitor therapy, often associated with favorable outcomes in melanoma, further supports the close biological relationship between melanocyte autoimmunity and antitumor immunity [53,54,55].

Taken together, the available evidence supports the view that HN is a highly informative immunological model situated at the intersection of autoimmunity, immune tolerance, pigmentary biology, and tumor immunology.

## 16. Future Directions

Future research should focus on defining the cellular and molecular architecture of HN across different evolutionary stages. Single-cell RNA sequencing, spatial transcriptomics, multiplex immunofluorescence, and T-cell receptor sequencing could clarify the relationships between CD8+ effector cells, regulatory T cells, PD-L1+ neutrophils, macrophages, dendritic cells, natural killer cells, and residual melanocytes. Particular attention should be given to the PD-1/PD-L1 axis. The discovery of PD-L1+ neutrophils in HN opens new questions regarding neutrophil recruitment, differentiation, survival, and functional plasticity. It remains unclear why some HNs exhibit neutrophil infiltration while others do not and whether this variability correlates with lesion stage, the probability of repigmentation, or the risk of vitiligo development [29].

Finally, clinical studies should clarify which patients with HN require autoimmune screening, dermoscopic follow-up, biopsy, or melanoma surveillance. Standardized criteria could improve management and reduce unnecessary excisions while maintaining diagnostic safety.

## 17. Conclusions

HN is a biologically complex condition that may represent a paradigm of controlled melanocyte autoimmunity. Based on current evidence, we propose that HN provides a useful model for studying how melanocyte-directed immune responses can be effectively activated and subsequently regulated. Its pathogenesis reflects a dynamic balance between cytotoxic immune activation and regulatory immune restraint. The study of HN provides valuable insights into immune tolerance, pigmentary autoimmunity, melanoma immunology, and tumor immunosurveillance, with important implications for future therapeutic strategies.

## Figures and Tables

**Table 1:** Histological differential diagnosis of halo nevus and related melanocyte-directed immune disorders.

Histopathological Feature	Halo Nevus (HN)	Regressing Melanoma	Melanoma-Associated Leukoderma (MAL)	Immunotherapy-Induced Vitiligo-like Reaction
**Underlying melanocytic lesion**	Benign melanocytic nevus	Melanoma	Absent	Absent
**Architectural symmetry**	Preserved	Lost/asymmetrical	Not applicable	Not applicable
**Cytological atypia**	Absent	Present	Absent	Absent
**Melanocytic maturation**	Preserved	Absent	Not applicable	Not applicable
**Pagetoid spread**	Absent	Frequently present	Absent	Absent
**Pattern of lymphocytic infiltrate**	Dense, diffuse, predominantly CD8+ T cells surrounding and infiltrating nevus nests	Variable, mixed CD4+/CD8+ infiltrate with regression-associated inflammation	Sparse superficial lymphocytic infiltrate	Mild superficial perivascular lymphocytic infiltrate
**Intensity of inflammatory infiltrate**	Marked	Variable	Mild	Mild
**Melanocyte loss**	Nevus cells and adjacent epidermal melanocytes	Partial or complete, associated with residual melanoma	Complete epidermal melanocyte loss	Complete or near-complete epidermal melanocyte loss
**Pigment incontinence/Melanophages**	Frequent	Frequent	Variable	Variable
**Fibrosis/Regression changes**	Minimal or absent	Frequent	Absent	Absent
**Regression-associated vascular changes**	Absent	Common	Absent	Absent
**Residual atypical melanocytic proliferation**	Absent	Present	Absent	Absent
**PRAME**	Negative	Positive	Negative	Negative
**Ki-67**	Low proliferative index	Increased	Negative	Negative
**Most useful diagnostic clue**	Symmetric benign nevus with preserved maturation and dense CD8+ infiltrate	Residual atypical melanocytes with architectural disorder and regression	Depigmented skin without melanocytic proliferation	Vitiligo-like inflammatory pattern in patients receiving immune checkpoint inhibitors

Abbreviations: HN, halo nevus; MAL, melanoma-associated leukoderma; PRAME, preferentially expressed antigen in melanoma.

## Data Availability

No new data were created or analysed in this study. Data sharing is not applicable to this article.
